# The impact of smoking on response to tumor necrosis factor-α inhibitor treatment in patients with ankylosing spondylitis

**DOI:** 10.55730/1300-0144.5661

**Published:** 2023-02-01

**Authors:** Handan YARKAN TUĞSAL, Gökçe KENAR, Gerçek CAN, Sedat ÇAPAR, Berrin ZENGİN, Servet AKAR, Ediz DALKILIÇ, Soner ŞENEL, Süleyman Serdar KOCA, Berna GÖKER, Ayten YAZICI, Nevsun İNANÇ, Hülya ELLİDOKUZ, Nurullah AKKOÇ, Fatoş ÖNEN

**Affiliations:** 1 Department of Internal Medicine, Division of Rheumatology, Faculty of Medicine, Dokuz Eylül University, İzmir, Turkiye; 2 Department of Statistics, Faculty of Medicine, Dokuz Eylül University, İzmir, Turkiye; 3 Division of Rheumatology, Department of Internal Medicine, Faculty of Medicine, Katip Çelebi University, İzmir, Turkiye; 4 Division of Rheumatology, Department of Internal Medicine, Faculty of Medicine, Uludağ University, Bursa, Turkiye; 5 Division of Rheumatology, Department of Internal Medicine, Faculty of Medicine, Erciyes University, Kayseri, Turkiye; 6 Division of Rheumatology, Department of Internal Medicine, Faculty of Medicine, Fırat University, Elazığ, Turkiye; 7 Division of Rheumatology, Department of Internal Medicine, Faculty of Medicine, Gazi University, Ankara, Turkiye; 8 Division of Rheumatology, Department of Internal Medicine, Faculty of Medicine, Kocaeli University, Kocaeli, Turkiye; 9 Division of Rheumatology, Department of Internal Medicine, Faculty of Medicine, Marmara University, İstanbul, Turkiye; 10 Department of Biostatistics and Medical Informatics, Faculty of Medicine, Dokuz Eylül University, İzmir, Turkiye; 11 Division of Rheumatology, Department of Internal Medicine, Faculty of Medicine, Celal Bayar University, Manisa, Turkiye

**Keywords:** Ankylosing spondylitis, smoking, tumor necrosis factor-alpha inhibitor, treatment response, registry

## Abstract

**Background/aim:**

To investigate the impact of smoking on disease activity, treatment retention, and response in patients with ankylosing spondylitis (AS) treated with their first tumor necrosis factor-α inhibitor (TNFi).

**Materials and methods:**

AS patients who started their first TNFi treatment for the active axial disease (BASDAI ≥ 4) from TURKBIO Registry were included. Treatment response of smoker (current and ex-smokers) and nonsmoker (never smoker) patients were primarily evaluated as achievement of BASDAI50 or improvement in BASDAI at least 20 mm at 3 months and 6 months compared to baseline.

**Results:**

There were 322 patients with AS (60% male, 59% smoker, mean age: 38.3 years). The median follow-up time was 2.8 years (Q1–Q3: 1.3–3.8), and disease duration was 3.5 years (Q1–Q3: 0.7–8.2). Smokers had male predominance (p < 0.001), lower ESR (p = 0.03), higher BASDAI (p = 0.02), BASFI (p = 0.05), HAQ-AS (p = 0.007), and ASDAS-CRP (p = 0.04) compared with nonsmokers at baseline.

In the multivariate analysis, male gender [OR 2.7 (95%CI 1.4–5), p = 0.002], and concomitant conventional synthetic disease-modifying antirheumatic drug use [OR 2.4 (95%CI 1.1–5.2), p = 0.03] were associated with better treatment response. There was an association of male gender [HR 2.4 (95%CI 1.6–3.7), p < 0.001], older age (≥30years) [HR 1.8 (95%CI 1.1–2.8), p = 0.01], and response to treatment [HR 1.8 (95%CI 1.2–2.9), p = 0.008] with better treatment retention. No impact of smoking status was found on treatment retention and response in univariate and multivariate analyses.

**Conclusion:**

This study suggested that smoking was associated with poorer patient-reported outcomes in biologic naïve AS patients initiating their first TNFi treatment, but it had no impact on the TNFi treatment response and retention rate.

## 1. Introduction

Cigarette smoking, one of the most severe public health problems, is a modifiable risk factor for several diseases. It is a major cardiovascular risk factor and estimated that 8 million deaths per year worldwide^1^. In the literature, a relationship between rheumatoid arthritis (RA) and smoking was first shown among inflammatory diseases, and smoking was reported to be the strongest known environmental risk factor for the development of RA [1]. Then, interest has been directed towards other rheumatic diseases to see whether smoking affects development, progression and treatment response in other rheumatic diseases. In patients with systemic lupus erythematosus, negative effects of smoking on the course of the disease and its treatment have been reported [2]. (The role of smoking in rheumatic diseases other than autoimmune spectrum (less female predominance and autoantibodies) is less clear. In terms of spondyloarthritis, a large population-based cohort study has shown that current smoking is significantly associated with incident ankylosing spondylitis (AS) [3]. Data from previous cross-sectional and longitudinal studies showed that smoking was an independent risk factor for structural damage in patients with axial spondyloarthritis (axSpA) [4–7]. However, its impact on global disease activity, functional status, and health-related quality of life in axSpA patients is unclear based on the limited data with contradictory results [8].

The tumor necrosis factor-α inhibitor (TNFi) drugs (adalimumab, certolizumab pegol, etanercept, golimumab, and infliximab) have revolutionized the treatment of AS over the past 20 years. Nevertheless, there are still a considerable number of patients who are resistant to treatments with TNFi. The response rates of *the Assessment of SpondyloArthritis International Society* (ASAS)20, ASAS40, ASAS partial remission were 58%–64%, 40%–47%, and 20%–23%, respectively in AS patients treated with the five TNFi drugs in the phase III trials [9–10]. Furthermore, real-life data showed that as many as 45% of patients with AS discontinued TNFi therapy within the first two years [11]. Older age, negative HLA–B27, and higher baseline disease activity were defined as the independent predictors of the nonresponse to first TNFi [12–14]. Furthermore, conflicting results had been reported in previous studies on the effect of smoking on the TNFi response [15].

The primary aim of this study was to investigate the impact of smoking on baseline disease activity, treatment response (BASDAI50/20) [9] and, treatment retention in Turkish patients with AS treated with their first TNFi therapy. We also aimed to determine the factors that could be associated with treatment efficacy and treatment retention, and the effect of smoking on treatment response evaluated by ASDAS [16].

## 2. Material and method

### 2.1 Patients

In this observational cohort study, we included adult AS patients who started their first TNFi treatment for active axial disease (BASDAI ≥ 4) and who had at least two visits after the initiation of therapy by the time of 1 January 2018 in the TURKBIO registry.

We excluded patients with baseline BASDAI < 4 or who initiated TNFi therapy for an indication other than axial disease, patients with <2 follow-up visits, patients who discontinued follow-up with no recorded reason, and patients with unknown smoking status.

TURKBIO registry is a nationwide biological database contributed by 14 different centers across Turkey. It includes adult (≥18 years) patients with RA, AS, nonradiographic axial spondyloarthritis (nr-AxSpA), and psoriatic arthritis. Demographic and clinical features, follow-up parameters related to disease and treatment, current and previous treatments, adverse events and discontinuation rates, and reasons were registered electronically using open-source software at each of the 3–6 months visits. The Drug Regulatory Authority of the Health Ministry of Turkey and the Dokuz Eylül University Ethics Committee has approved the Registry Project as a phase IV observational study. The whole study was performed according to the Declaration of Helsinki and the ethical approval for the secondary use of the data included in the TURKBIO has been obtained previously (Dokuz Eylül University Ethics Committee; 13.04.2017; protocol no: 304-SBKAEK). Patients signed a written informed consent form before their inclusion in the study. We followed the STROBE checklist of items for reports of cohort studies.

### 2.2 Smoking status

Smoking was defined as using >1 cigarette/day without reference to the quantity (e.g., pack-years). Ex-smoker was defined as one who had smoked more than 100 cigarettes in the lifetime but has not smoked in the last 28 days. Patients’ smoking status at the start of TNFİ therapy was obtained from the data of the closest yearly visit in the TURKBIO registry. They were stratified into two groups: 1. Smokers (including current- and ex-smokers) and 2. Nonsmokers (including never-smokers).

### 2.3 Disease activity, function, mobility and health-related quality of life

Disease activity was evaluated by the Bath ankylosing spondylitis disease activity index (BASDAI) [17] and, the ankylosing spondylitis disease activity score calculated using C-reactive protein (ASDAS-CRP) [18]. Functional status and mobility were assessed using the Bath ankylosing spondylitis functional index (BASFI) [19] and the Bath ankylosing spondylitis metrology index (BASMI) [20], respectively. Patients completed the health assessment questionnaire for ankylosing spondylitis (HAQ–AS) for health-related quality of life assessment [21].

### 2.4 Treatment response

Clinical response to TNFi was primarily evaluated by BASDAI50/20 [improvement of at least 50% in the BASDAI score or an absolute change of 20 units (on a 0 to 100 scale) after three months of therapy] as recommended by ASAS [9]. Patients who achieved clinical response at both third- and sixth-months’ visits compared to baseline were defined as responders. The patients with a clinical response at either the third- or sixth-months’ visits were also classified as ‘responders’ when there was a reported reason for missing visits. If there were no visits within the given month, the closest visit was chosen not exceeding eight weeks. For baseline visit, the period between one month before and one week after the TNFi therapy were allowed.

Furthermore, ASDAS clinical responses [16] as ASDAS-LDA (Low Disease Activity, ASDAS 1.3–2.1), ASDAS CII (Clinically Important Improvement, Δ ≥ 1.1), and ASDAS MI (Major Improvement, Δ ≥ 2) were evaluated at third and sixth months of therapy.

### 2.5 Treatment retention

Treatment retention was defined as the number of years from the initiation to the end of therapy. Treatment cessation for any reason (e.g., local reactions, infections, surgery) less than two months were allowed.

The reasons for treatment discontinuation were recorded in TURKBIO as lack of effect, adverse events, cancer, follow-up at other centers, pregnancy, infections, discontinuation of follow-up, death, surgery, disease remission, patient preference, and other reasons. For this study, the reasons for discontinuation were collected into three groups; adverse events (including infection, death, and cancer), lack of effect, and others. The patients without a record of the reason for treatment cessation were excluded from the study.

### 2.6 Statistics

Demographic and descriptive data are presented as median, first and third quartile (Q1–Q3). Baseline characteristics in categorical variables were compared between smokers (ever or current) and nonsmokers using nonparametric tests (χ^2^, Kruskal Wallis and Mann Whitney tests). In all tests, a 5% type-1 error level was used to infer statistical significance.

The univariate analyses to identify variables associated with treatment response were investigated using Pearson’s chi-square, Fisher exact, Student’s t, and Mann-Whitney U tests, where appropriate. Age (<30 years old vs. ≥30 years old) and disease duration (<4 years vs. ≥4 years) were included as categorical variables to allow for possible nonlinear effects. For the multivariate analysis, the possible factors [age, concomitant conventional synthetic disease-modifying antirheumatic drug (csDMARD) use, and gender] identified with univariate analyses were further entered into the logistic regression analysis to determine independent predictors of treatment response. The odds ratios (OR) and 95% confidence intervals (95% CI) were also calculated. In the regression model, both age and gender were retained as covariates to control their influence on other covariates. Since baseline BASDAI were higher in smokers, parameter estimates from the multivariable model were adjusted for baseline difference. Hosmer-Lemeshow goodness of fit statistics was used to assess model fit.

The effect of smoking and other factors on treatment retention was investigated using the log-rank test. The Kaplan-Meier survival estimates were calculated. The possible factors identified with univariate analyses (age, gender, disease duration, and response to treatment) were further entered into Cox regression analysis, with backward selection, to determine independent predictors of adherence and calculated hazard ratios (HRs). The effects of potential covariates (age and gender) on treatment retention were adjusted by retaining them in the multivariable model. However, adjustment for baseline BASDAI was performed since smokers have higher baseline BASDAI.

Stratified analyses were performed according to gender.

## 3. Results

### 3.1 Baseline characteristics

There were 322 patients (60% male, mean age: 38.3 years) who fulfilled the modified New York classification criteria for AS [22] and followed up since the first TNFi therapy in TURKBIO by 2018. Among the 322 patients, 191 (59%) were smokers (125 current- and 66 ex-smokers) and 73% of smokers were male.

At baseline, smoker patients had higher BASDAI, BASFI, HAQ-AS, ASDAS-CRP, and lower erythrocyte sedimentation rate (ESR) than nonsmokers. Median BASMI was slightly higher in smokers than nonsmokers but statistically nonsignificant (p = 0.34). There were no differences concerning age, disease duration, HLA-B27 status, serum CRP levels, BMI measurements, medications including csDMARDs, and the reasons for stopping TNFi treatment between smoker and nonsmoker patient groups (Table 1).

### 3.2 Treatment response

Among 322 AS patients, 267 (83%) responded to the first TNFi therapy based on BASDAI50/20. Smoker and nonsmoker patients had similar treatment responses (BASDAI50/20: 85% and 79%, respectively). There was no statistically significant difference between current vs. never smokers (p = 0.19), current vs. ex-smokers (p = 0.94), and current vs. never+ ex-smokers (p = 0.31) regarding TNFi response. When men and women were analyzed separately, TNFi response was not different between smoker vs. nonsmoker (p = 0.98, p = 0.71), current vs. never-smoker (p = 0.90, p = 0.47), current vs. ex-smoker (p = 0.73, p = 0.38), or current vs. never+ ex-smoker (p = 0.81, p = 0.40) groups in both men and women respectively.

There was no statistically significant difference between smoker and nonsmoker patients regarding ASDAS-CII (p = 0.32) and ASDAS-MI response (p = 0.65) and achievement of ASDAS-LDA (p = 0.31) at both 3 and 6 months. ASDAS-CII (p = 0.75, p = 0.83) and ASDAS-MI response (p = 0.25, p = 0.80), and achievement of ASDAS-LDA (p = 0.37, p = 0.62) were also not different between smoker and nonsmoker patients in both men and women respectively.

### 3.3 Change in the follow-up outcomes

Third and 6th-month improvement in HAQ-AS was significantly better in nonsmokers than smokers. However, improvement in the other follow-up parameters (ΔBASDAI, ΔBASFI, ΔBASMI, and ΔASDAS-CRP) at 3 and 6 months was not different between the groups. When males and females were analyzed separately, nonsmoker males had better improvement in third month BASFI and HAQ-AS and 6th-month HAQ-AS than smoker males. However, the improvement of HAQ-AS at third and 6th-month visits was not different between smoker and nonsmoker females.

The possible factors affecting treatment response, including gender, concomitant csDMARD use, and age (grouped as <30 and ≥30) in the univariate analysis, were further entered into the logistic regression analysis. Multivariate regression analysis showed that males [OR: 2.7; (95% CI 1.4–5), p = 0.002] and concomitant csDMARD users [OR: 2.4; (95% CI 1.1–5.2) p = 0.03] had better response rates of BASDAI50/20 (Table 2).

### 3.4 Treatment retention

The total follow-up time was 908.4 patient years. The median follow-up time was 2.8 years (Q1–Q3: 1.3–3.8), and ever-smokers had followed longer [2.9 years (Q1–Q3: 1.5–4.3)] than never smokers [2 years (Q1–Q3: 1.1–3.5)].

Smokers had similar drug survival [2.36 (1.00–3.72) years] to nonsmokers [1.75 (0.77–3.22) years; Kaplan Meier log rank = 0.06)] (Figure). Both female smokers [1.6 (0.5–2.9) years] and male smokers [2.5 (1.3–4.1) years] had similar drug survival to female nonsmokers [1.4 (0.5–2.9) years] and male nonsmokers [2.6 (1–3.6) years] (both Kaplan Meier log rank = 0.7) respectively.

The possible factors affecting drug survival in univariate analysis were gender, age (≥30 years old), response to treatment, and disease duration (≥4 years). In female patients, age (≥30 years old), disease duration (≥4 years), and response to TNFi therapy, in males age (≥30 years old) were the possible factors affecting drug survival determined by univariate analysis.

Multivariate analysis showed that treatment retention was better in men [HR: 2.4, (95% CI 1.6–3.7), p < 0.001], patients who were older than 30 years of age [HR: 1.8, (95% CI 1.1–2.8), p = 0.01] and treatment responders [HR: 1.8, (95% CI 1.2–2.9) p = 0.008] (Table 3).

## 4. Discussion

This study showed that smoking was associated with higher baseline disease activity, decreased function, and impaired health-related quality of life in biologic naïve AS patients initiating their first TNFi in the TURKBIO registry. However, it had no impact on their first TNFi treatment response and drug survival.

Several studies reported higher baseline disease activity, functional disability, and impaired quality of life in smoker AS patients than nonsmokers, similar to our study [7,8,23–28]. Mattey et al. showed the negative and dose-dependent impact of smoking on measures of disease severity in AS patients and was independent of age, gender, deprivation level, and disease duration [25]. Another report from the Scotland Registry for AS suggested that smoking cessation was associated with lower disease activity and better physical function and quality of life in patients with AS [27].

Evidence suggests that smoking is associated with axial skeletal radiographic severity and disease progression over time in ax-SpA patients [5, 7, 29–31]. A study in the OASİS cohort, Ramiro et al., showed that the impact of smoking on radiographic progression was through impairment in disease activity in patients with AS [30]. Another study suggested a strong association between the amount of smoking and spinal radiographic progression evaluated by mSASSS in AS patients [32].

There is limited data with conflicting results regarding the impact of smoking on TNFi response in AS patients [33]. Two studies with adjusted analysis reported its negative impact on the TNFi treatment efficacy. The first study revealed that current and ex-smoker AS patients were less likely to achieve BASDAI50/20 response than nonsmokers at 3 and 6 months in the DANBİO cohort, including 1425 patients [34]. Current smokers had 46% lower odds of achieving BASDAI50/20. Ciurea et al. also showed that current smokers had poorer BASDAI50 and ASDAS response s rates than nonsmokers among patients with elevated baseline CRP. However, there was no lower response rate in smokers with normal serum CRP levels [35]. Furthermore, the difference in BASDAI change over time (ΔBASDAI) was not clinically or statistically significant, despite current smokers having 54% lower odds of BASDAI50 at one year.

No statistically significant differences were found in studies investigating change in continuous outcomes of follow-up with TNFi between smoker and nonsmoker ax-SpA patients [33, 36, 37]. Kydd et al. showed that smoking did not affect TNFi response in terms of health-related quality of life among Australian AS patients [37]. Recently Zhao et al. also reported that response to TNFi drugs did not differ according to smoking status in the British AxSpA cohort [33]. In our study, TNFi response investigated by several follow-up parameters, including both continuous or binary outcomes and drug survival were not different between smoker and nonsmoker AS patients. Only HAQ-AS score improvement at 3 and 6 months was found to be better in nonsmokers than smokers.

Contradictory results between the studies may be related to using continuous or binary response outcomes. Since smokers had higher baseline disease activity than nonsmokers, they would be less likely to reach a binary response, although they had similar absolute improvement in disease activity or other follow-up parameters over time [33]. On the other hand, better response rates could be expected in smoker AS patients with higher disease activity since higher baseline disease activity was associated with better clinical response in patients with AS initiating TNFi.

In our study, males, and active csDMARD users had better response rates. Male gender was reported as a baseline predictor of TNFi response in previous studies, consistent with our study [38].

To date, the impact of smoking on TNFi response was investigated primarily in observational database studies with different methodological features and significant limitations due to their retrospective designs. The criteria used for either inclusion or exclusion of patients also may lead to essential differences in the results. In this study, we excluded patients with baseline BASDAI < 4, patients who initiated TNFi for nonaxial involvements, and those with follow-up visits less than two (without known cause) to provide a homogenous group and evaluate clinical response more accurately. All these are the strengths of our study, although they led to a decrease in the number of patients as a limitation.

The definition of treatment success is another issue. Some studies define response by continuous variables (Etc. BASDAI, ASDAS), others by dichotomized variables as responder or nonresponder. Our study used binary BASDAI50/20 response as the primary outcome and evaluated other outcomes, including ASDAS-LDA, ASDAS-CII, and ASDAS-MI. Furthermore, changes at 3 and 6 months from baseline in all these parameters were investigated to see absolute improvements. We consider that the main strength of this study was the exact definition of clinical response to TNFi treatment.

One of the main limitations of this study was not to include smoking duration and intensity to evaluate whether there was a dose-dependent effect. In addition, potential confounders such as the socioeconomic and exercise status of the patients were not included in our database.

In conclusion, our findings suggested that although the baseline disease activity was higher in smoker AS patients than nonsmokers, smoking had no impact on their first TNFi responses. Based on contradictory results in the literature, there is a need to investigate the association between smoking and response to TNFi in prospective studies that evaluated the effects of the amount and duration of smoking and its cessation on treatment. However, irrespective of the results of the studies, smoking cessation should be strongly recommended in all AS patients because of its negative effects on disease activity and radiographic progression and well-known risk for several comorbidities, including cardiovascular and chronic lung diseases.

## Figures and Tables

**Figure:**
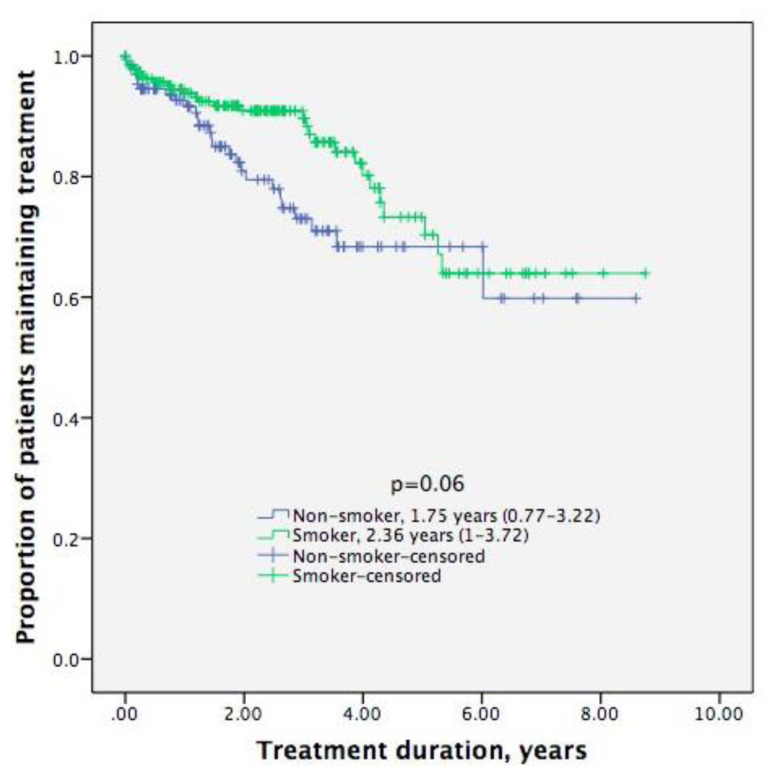
Treatment retention according to smoking status results from Kaplan-Meier analysis [median (95% CI)].

**Table 1:** Demographics and patient characteristics.

	Smoking status		

	Smoker n = 191	Nonsmoker n = 131	p

Age, median (Q1–Q3), years	38 (30–44)	39 (30–46)	0.397

Disease duration, median (Q1–Q3), years	3.3 (0.6–8.9)	3.7 (0.9–7.3)	0.795

Male, n (%)	139 (72)	56 (42)	**<0.001**

HLA positive^a^, n (%)	95 (69)	58 (62)	0.228

Body Mass Index^b^, kg/m^2^, median (Q1–Q3)	25.1 (22.6–29.4)	27.1 (23.9–29.5)	0.142

CRP, mg/L, median (Q1–Q3)	11.5 (5–25.2)	13 (5–29)	0.772

ESR, mm/h, median (Q1–Q3)	26 (12–42.2)	32 (19–49)	**0.029**

Baseline disease characteristics, median (Q1–Q3)			

BASDAI	58 (49–66)	54 (46–62)	**0.019**

BASFI	38 (25–52)	34 (21.25–45.5)	**0.052**

BASMI	20 (4–50)	15 (4–30)	0.338

HAQ-AS	0.75 (0.5–1)	0.625 (0–0.875)	**0.007**

ASDAS-CRP	3.7 (2.7–4.2)	3.35 (0–3.975)	**0.042**

TNFi type, n (%)			0.315

Adalimumab	39 (20)	33 (25)	

Etanercept	57 (30)	31 (24)	

Infliximab	58 (30)	32 (24)	

Golimumab	26 (14)	25 19)	

Certolizumab	11 (6)	10 (8)	

Current DMARD use	29 (15)	17 (13)	
Methotrexate, n (%)	16 (8)	6 (5)	0.492
Sulphasalazine, n (%)	13 (7)	11 (8)	

Previous DMARD use, n (%)	18 (10)	15 (11)	0.556

Methotrexate	7 (4)	6 (4.5)	

Sulphasalazine	9 (5)	6 (4.5)	

Reason of treatment discontinuation^c^ n (%)	67 (35)	37 (28)	0.588

Advers events	8 (4)	6 (5)	

Lack of efficacy	25 (13)	16 (12)	

Other	34 (18)	15 (11)	

Total follow-up time, median (Q1–Q3), years	2.9 (1.5–4.3)	2 (1.06–3.5)	**0.003**

Treatment response^d^, n (%)	163 (85)	104 (79)	0.163

Changes at 3^rd^ months^e^, median (Q1–Q3)			

BASDAI	4.4 (3.5–5.6)	4.2 (3–5.3)	0.278

BASFI	2.2 (0.4–3.7)	2.1 (0.7–3.5)	0.809

BASMI	1 (–0.4–4.8)	0.4 (–0.4–3)	0.287

ASDAS-CRP	2.2 (1.4–3.1)	2.1 (0.7–3.3)	0.282

HAQ-AS	0.37 (−0.1–0.7)	0.62 (0.25–0.87)	**0.006**

Changes at 6^th^ months^e^, median (Q1–Q3)			

BASDAI	4.4 (3.7–5.5)	4.4 (3.6–5.5)	0.817

BASFI	2.4 (2.7–4)	2.4 (1–3.8)	0.683

BASMI	3 (0.4–5.2)	1 (0.4–3)	0.108

ASDAS-CRP	2.5 (0.8–3.3)	2.2 (−0.05–3.3)	0.437

HAQ-AS	0.5 (−0.05–0.75)	0.6 (0.20–0.99)	**0.055**

Abbreviations: ASDAS-CRP: Ankylosing Spondylitis Disease Activity Score calculated using C-Reactive Protein; BASDAI: Bath Ankylosing Spondylitis Disease Activity Index; BASFI: Bath Ankylosing Spondylitis Functional Index; BASMI: Bath Ankylosing Spondylitis Metrology Index; DMARD: Disease-Modifying AntiRheumatic Drug; HAQ-S: Health Assessment Questionnaire for Ankylosing Spondylitis; TNFi: Tumor necrosis factor-α inhibitor; Q1–Q3: First and third quartile

a Not all patients had HLA B27 status, HLA B27 was available for 231 patients. Percentages were reported as positivity among known HLA status.

b Not all patients had BMI, analyses were done with 193 patients.

c Percentages of patients who have stopped TNFi treatment according to smoking status.

d Treatment response was defined as BASDAI50/20 mm response both at 3^th^ and 6^th^ months’ visits compared to baseline.

e Changes at 3^rd^ and 6^th^ month mean decreases from baseline

**Table 2:** Results of multivariate logistic regression analysis for factors affecting treatment response.

Variable	OR (95% CI)	P-value
Gender (male vs. female)	2.7 (1.4–5)	0.002
csDMARD use (yes vs. no)	2.4 (1.1–5.2)	0.033
Age (≥30 vs. <30 years old)	1.4 (0.6–3.1)	0.372

Abbreviations: csDMARD: conventional synthetic Disease-Modifying AntiRheumatic Drug CI: Confidence Interval OR: Odds ratio

Parameter estimates from the multivariable model were adjusted for baseline BASDAI

**Table 3:** Results of multivariate cox regression analysis for factors affecting treatment retention.

Variable	HR (95% CI)	P-value
Gender (male vs. female)	2.4 (1.6–3.7))	<0.001
Age (≥30 vs. <30 years old)	1.8 (1.1–2.8)	0.012
Disease duration (≥4 vs. <4 years)	1.5 (0.9–2.2)	0.072
Treatment response (yes vs. no)	1.8 (1.2–2.9)	0.008

Abbreviations: CI: Confidence Interval HR: Hazard ratio

Parameter estimates from the multivariable model were adjusted for baseline BASDAI

## Footnotes

1 World Health Organization (2019). Tobacco fact sheet [online]. Website https://www.who.int/news-room/fact-sheets/detail/tobacco [accessed 24 May 2022].
